# Supplementary figures and images for: Study of TRAF3IP3 for prognosis and immune infiltration in hepatocellular carcinoma
Source: PeerJ. 2024 Dec 12;12:e18538. doi: 10.7717/peerj.18538 (PMC11646420; doi:10.7717/peerj.18538)

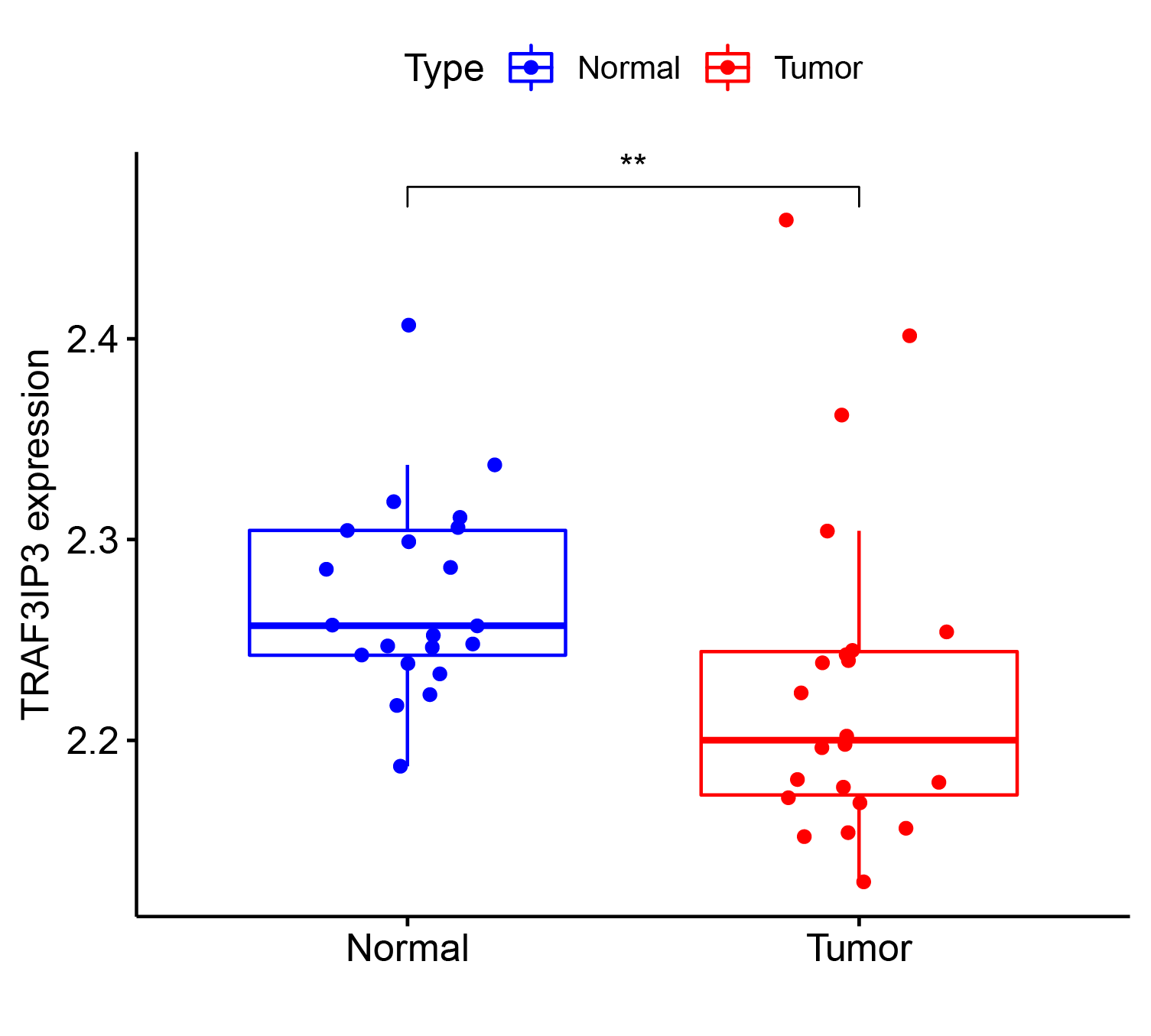

Supplement: Supplemental Information 1 [file peerj-12-18538-s001.png]

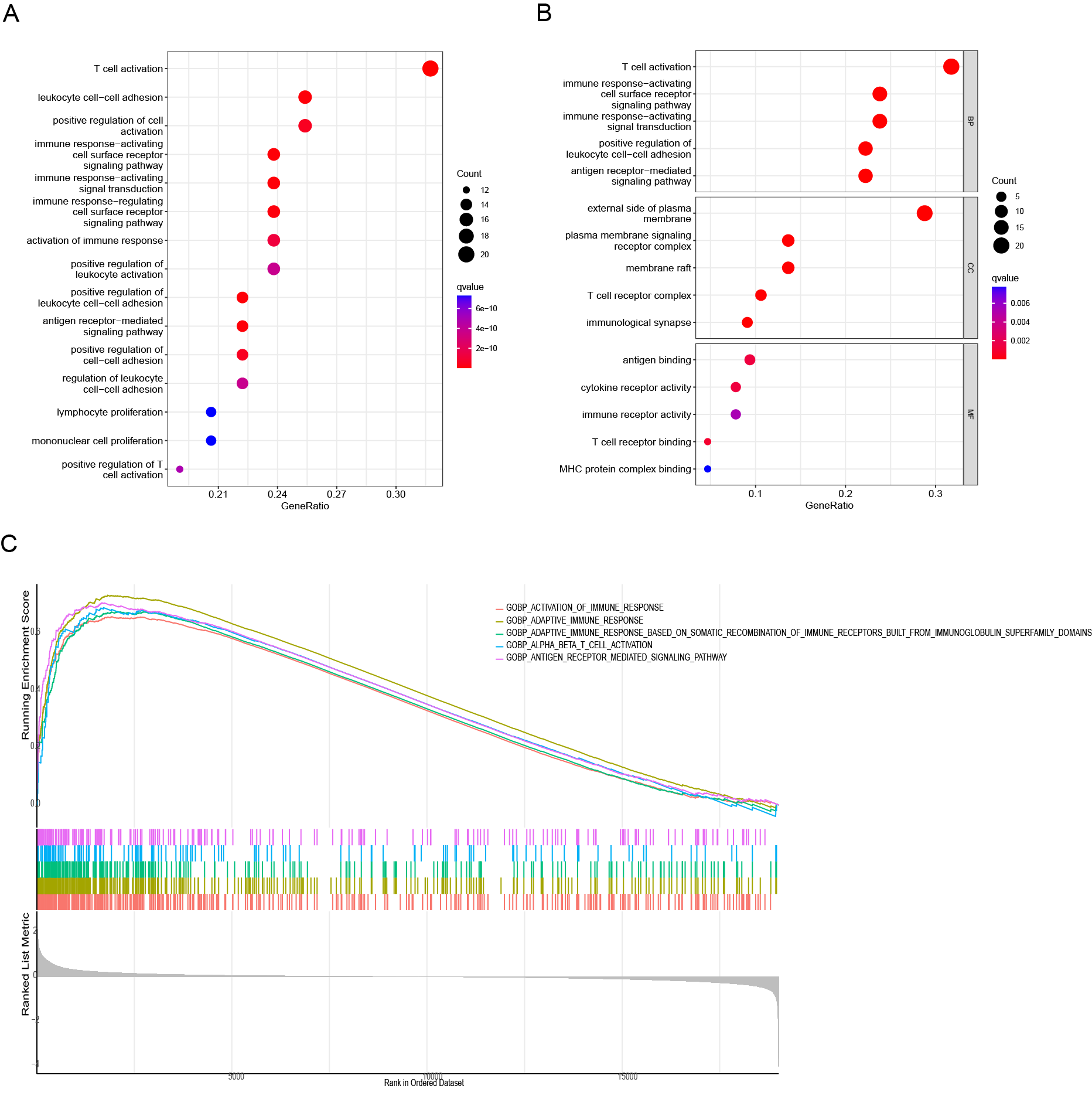

Supplement: Supplemental Information 2 — (A) GO and (B) KEGG analyses of DEGs in samples with low and high TRAF3IP3 expression. (C) Enrichment of genes in the typical pathways by GSEA function analysis in HCC. [file peerj-12-18538-s002.png]

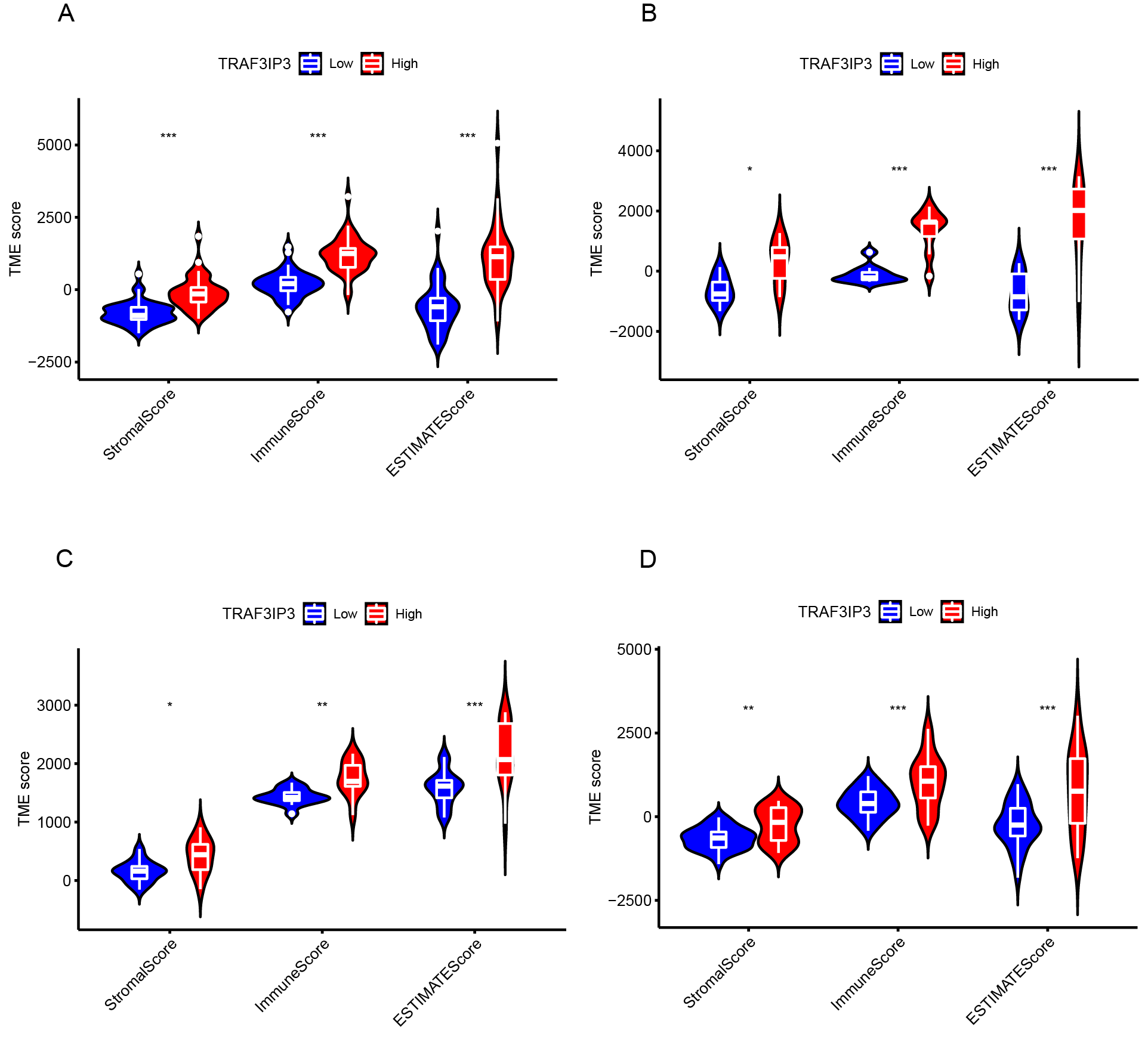

Supplement: Supplemental Information 3 — Comparison of stroma score, immune score, and estimate score between TRAF3IP3 high and low expression groups from external cohorts (A, GSE121248, n = 107; B, GSE60502, n = 36; C, GSE76427, n = 167; D, GSE102451, n = 59). [file peerj-12-18538-s003.png]

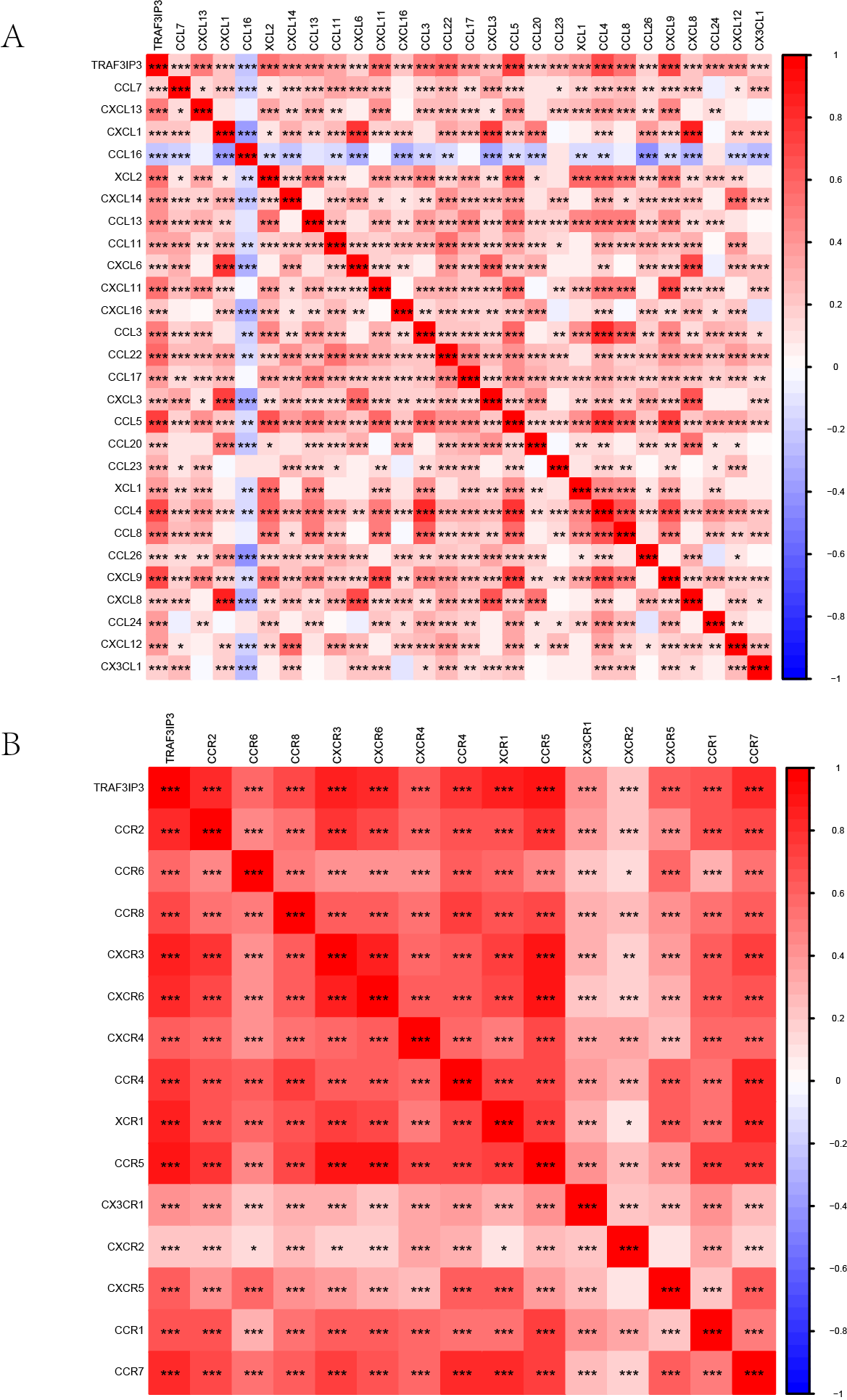

Supplement: Supplemental Information 4 [file peerj-12-18538-s004.png]

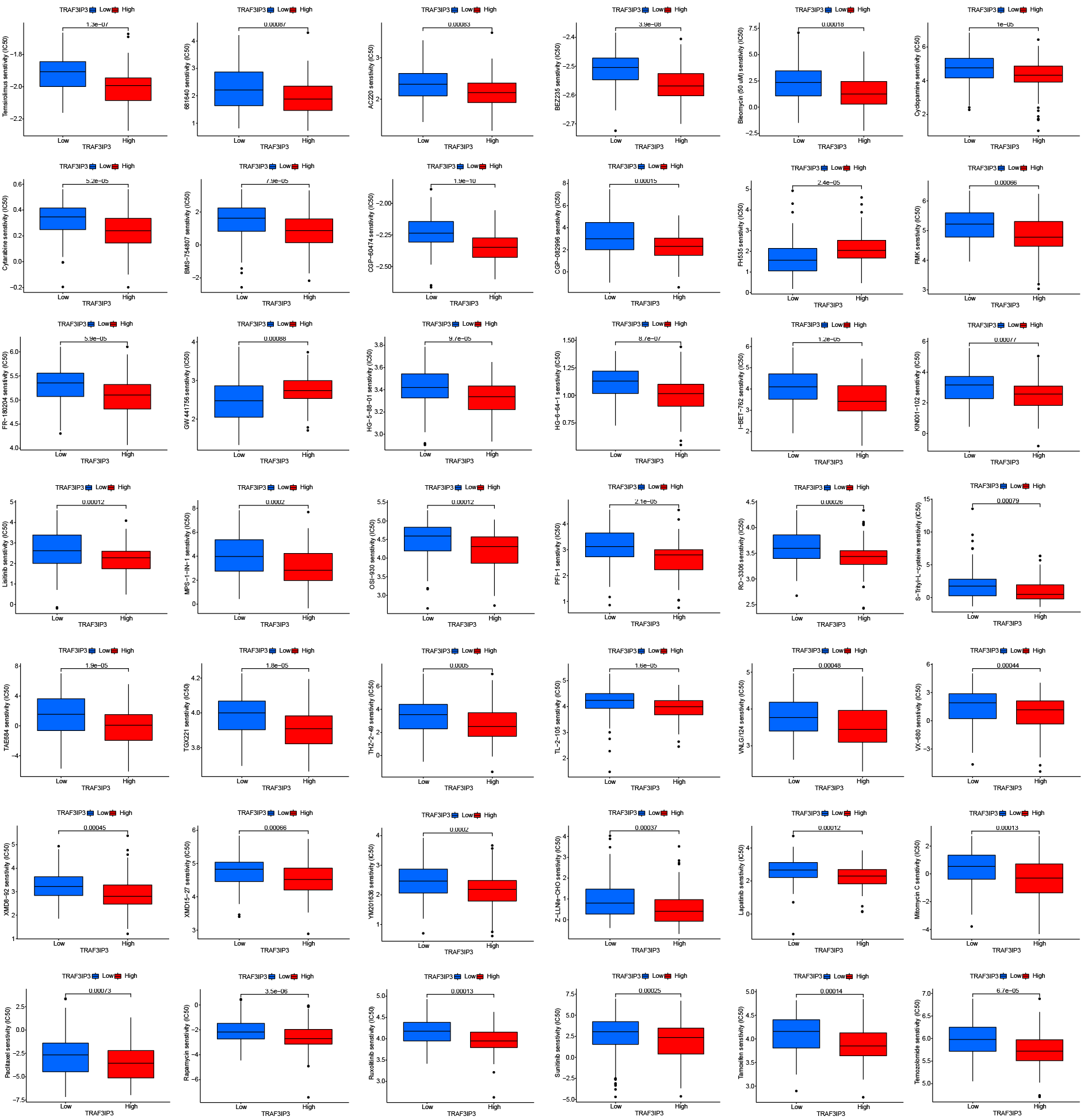

Supplement: Supplemental Information 5 [file peerj-12-18538-s005.png]

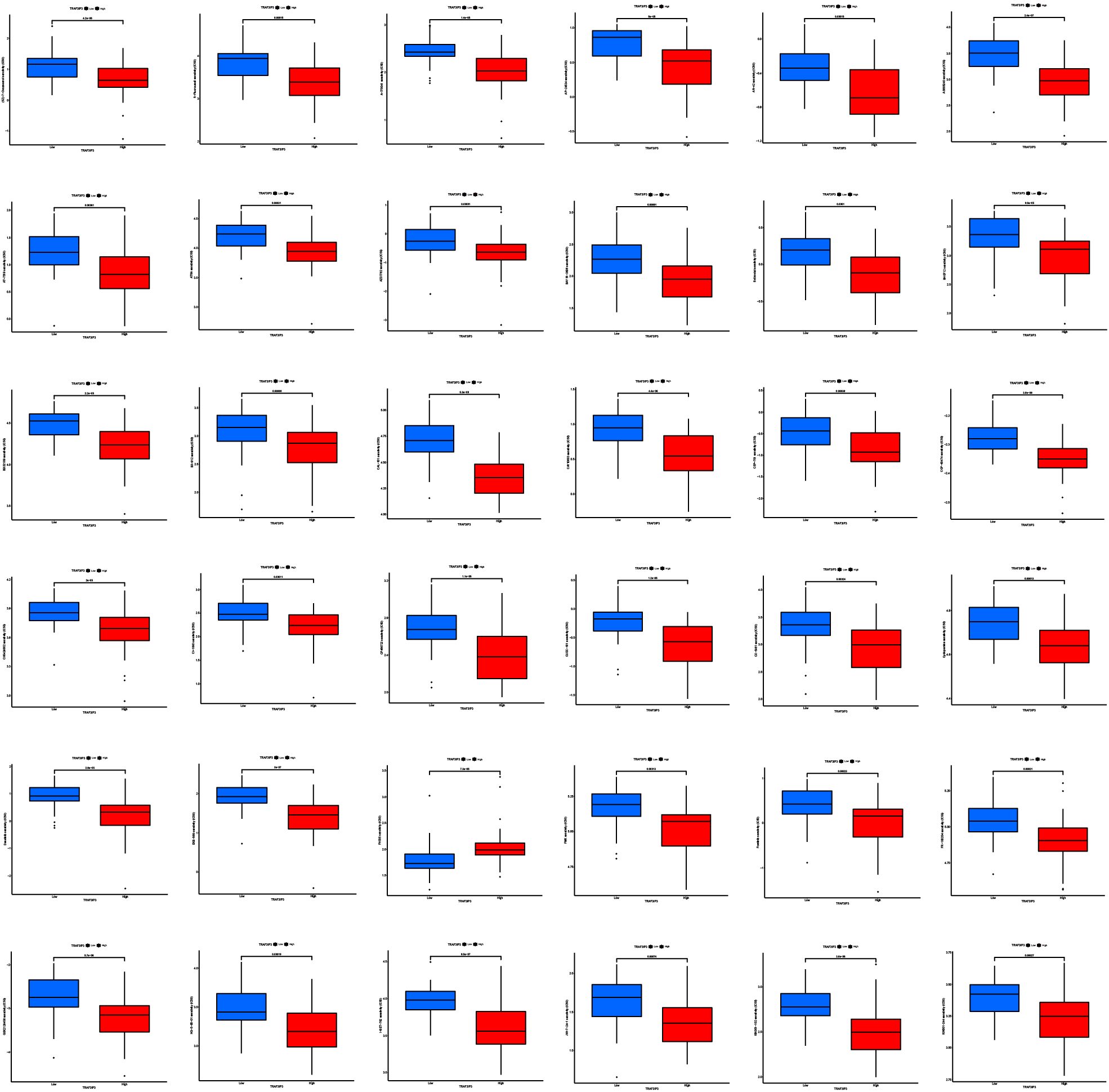

Supplement: Supplemental Information 6 [file peerj-12-18538-s006.png]

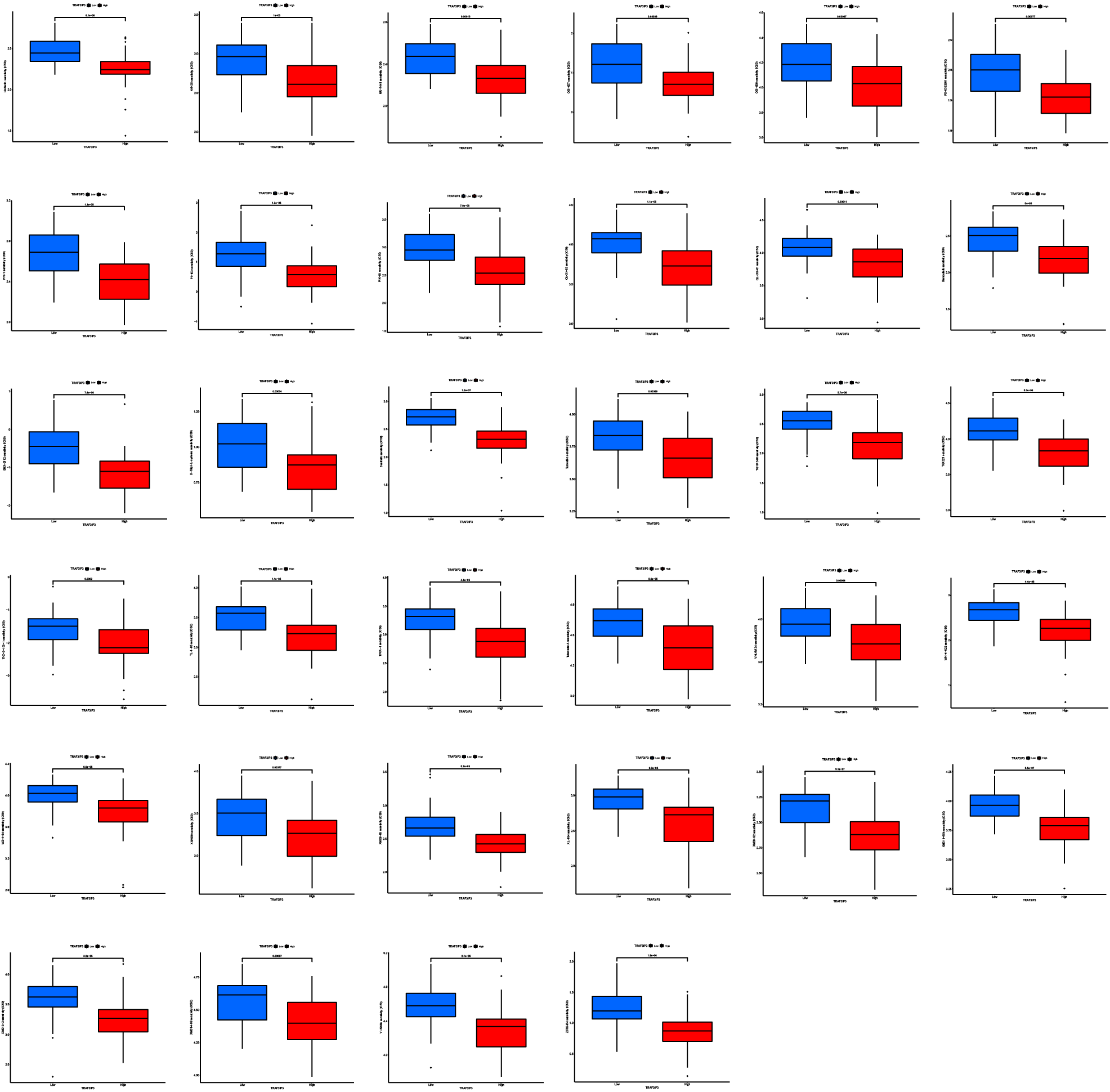

Supplement: Supplemental Information 7 [file peerj-12-18538-s007.png]
